# Integrative Analysis of Genes Involved in the Global Response to Potato Wart Formation

**DOI:** 10.3389/fpls.2022.865716

**Published:** 2022-06-29

**Authors:** Lang Yan, Yan Li, Yuan Qing, Xiang Tao, Haiyan Wang, Xianjun Lai, Yizheng Zhang

**Affiliations:** ^1^Panxi Crops Research and Utilization Key Laboratory of Sichuan Province, College of Agricultural Science, Xichang University, Liangshan, China; ^2^College of Life Sciences, Sichuan Normal University, Chengdu, China; ^3^Sichuan Key Laboratory of Molecular Biology and Biotechnology, College of Life Sciences, Sichuan University, Chengdu, China

**Keywords:** potato, *Synchytrium endobioticum*, transcriptional profiling, gene coexpression networks, susceptible response

## Abstract

*Synchytrium endobioticum*, the causal agent of potato wart disease, poses a major threat to commercial potato production. Understanding the roles of transcriptionally regulated genes following pathogen infection is necessary for understanding the system-level host response to pathogen. Although some understanding of defense mechanisms against *S. endobioticum* infection has been gained for incompatible interactions, the genes and signaling pathways involved in the compatible interaction remain unclear. Based on the collection of wart diseased tubers of a susceptible cultivar, we performed phenotypic and dual RNA-Seq analyses of wart lesions in seven stages of disease progression. We totally detected 5,052 differentially expressed genes (DEGs) by comparing the different stages of infection to uninfected controls. The tendency toward differential gene expression was active rather than suppressed under attack by the pathogen. The number of DEGs step-up along with the development of the disease and the first, third and seventh of the disease stages showed substantially increase of DEGs in comparison of the previous stage. The important functional groups identified *via* Gene ontology (GO) and KEGG enrichment were those responsible for plant-pathogen interaction, fatty acid elongation and phenylpropanoid biosynthesis. Gene coexpression networks, composed of 17 distinct gene modules that contained between 25 and 813 genes, revealed high interconnectivity of the induced response and led to the identification of a number of hub genes enriched at different stages of infection. These results provide a comprehensive perspective on the global response of potato to *S. endobioticum* infection and identify a potential transcriptional regulatory network underlying this susceptible response, which contribute to a better understanding of the potato–*S. endobioticum* pathosystem.

## Introduction

Potato wart disease, at present the most severe quarantine disease affecting cultivated potato production worldwide, is caused by the soilborne obligate biotrophic fungus *Synchytrium endobioticum* (Schilb.) Percival (Curtis, 1921; Hampson and Coombes, 1985). The potato host cell greatly enlarges and surrounding cells divide irregularly, resulting in wart-like malformations and a nutrient sink (Hampson and Coombes, 1985). The tumor-like tissue of the wart progressively increases in size at the expense of the tubers, leading to unmarketable tubers and complete yield losses (Hampson, 1993). *Synchytrium endobioticum* exhibits a life cycle with a haploid sorus. Summer sporangia release several hundred zoospores that infect new tissue and transform under unfavorable conditions into diploid zygotes, resting or winter sporangia that penetrate host tissue and release to the soil when the host tissue decays (Busse et al., 2017). Because resting sporangia can survive and remain viable and infectious in the soil for decades, the major problem in current potato production is the contamination of the soil. There are no effective chemical control agents for eradicating the pathogen from contaminated soil (Obidiegwu et al., 2014). The only strategies to confine the disease are strict quarantine and phytosanitary measures as well as cultivation of resistant cultivars (De Boer, 2001; Flath et al., 2014; Obidiegwu et al., 2014; Przetakiewicz, 2015).

Plant–fungi interactions have evolved over millions of years, resulting in complicated mechanisms on both incompatible and compatible interaction (Asai and Shirasu, 2015; Cook et al., 2015; Wang and Wang, 2018). Although resistance and susceptibility are opposite sides of the same coin, most studies have focused for a long time on the resistance side in search for plant resistance genes (R-genes) and other defense genes (Pavan et al., 2010). Systematic studies on resistance of potato cultivars to *S. endobioticum* started a century ago. Conventional breeding schemes were successful in developing resistant varieties early in the twentieth century while are currently challenged by *S. endobioticum* pathotypes evolving and the increased risk of dissemination by potato tuber trade (Baayen et al., 2006; Obidiegwu et al., 2014). Several quantitative resistance loci for *S. endobioticum* resistance have been identified, in which the first one was a single dominant gene *Sen 1* located on potato chromosome IX, bringing resistance to pathotype 1(D1) through the recognition of the pathogen effector AvrSen1 (Hehl et al., 1999; Brugmans et al., 2006; van de Vossenberg et al., 2019). Based on two mapping populations, the quantitative resistance locus (QRL) *Sen2/6/18* on chromosome I were identified, which expressed resistance to pathotypes 2(G1), 6(O1), and 18(T1) respectively (Ballvora et al., 2011). Recently, newly locus *Sen2* located on chromosome XI which provides resistance to at least seven various virulent pathotypes of *S. endobioticum* were reported (Plich et al., 2018).

Although some understanding of defense mechanisms against *S. endobioticum* infection has been gained for incompatible interactions, including identification of several *Sen* loci conferring qualitative or quantitative potato resistance, the genes and signaling pathways involved in the compatible interaction remain unclear, especially the molecular basis of the induction of neo-plastic growth by this fungus. Insight into the molecular basis of plant disease susceptibility can be in further applied in breeding for resistance against a wide spectrum of pathogens. Besides the employment of multiple resistance genes in resistance breeding, knocking out (down) key susceptible genes could be an alternative method to develop cultivars with durable resistance. For example, the molecular responses of a susceptible wheat to *Fusarium graminearum* infection fit over the grain development processes depicted new clues to the understanding of effector-triggered susceptibility in plant–pathogen interactions (Chetouhi et al., 2016). Several host genes necessary for pathogen growth and infectious cycle were reported in other pathosystems (Pavan et al., 2010; Jia et al., 2011). A loss of functional mutations of such genes has already been successful in providing durable and broad-spectrum plant resistance, making the susceptibility genes (S-genes) a promising source of resistance in breeding strategies.

Although increasing knowledge is available, identifying S-genes still requires a better understanding of the molecular determinism of the plant-pathogen interacting system, including genome-wide approaches. Given the rapid development of high-throughput sequencing technology, RNA-Seq has been widely used to explore genome-wide gene expression patterns in compatible interaction (Tremblay et al., 2010; Tan et al., 2015). Although differential gene expression analyses have been carried out comparing transcription profiles, most of them provide a limited picture of the whole infection dynamics, prioritizing either on different stages of disease establishment or on organ-specific responses. In this study, we combined transcriptome and gene coexpression network (GCN) analyses to characterize regulated genes and signaling pathways in potato tuber tissue underlying global responses to *S. endobioticum* infection at the transcriptional level. GCNs constructed from gene expression data through the calculation of pairwise correlation coefficients could enrich genes’ causal susceptibility and correlate with certain immune responses in modules, representing well-defined biological interactions deduced by differentially expressed genes (DEGs). Also, GCNs helps in noise reduction by eliminating non-responding genes during pathogen attacks, providing better insights into molecular mechanisms of the host response during wart formation and pathogenesis. Comparing patterns of gene expression among samples with varying stages of infection highlights some of the potential genes in biological pathways leading to susceptibility, which could help explain the compatible interactions in the potato–*S. endobioticum* pathosystem. Identifying key functional genes and pathways in susceptible responses will ultimately be powerful for developing diagnostic markers, which may aid in the identification of novel pathotype-specific effector genes and the development of new interventions against this pathogenic fungus.

## Materials and Methods

### Plant Collection and Disease Assessment

The potato cultivar Qingshu 9, which is susceptible to potato wart disease, was grown in a field affected by potato wart disease the previous year. The field was located in Puge county, Liangshan, in southwestern China (E102°41′, N26°64′, elevation: 2,521 m a.s.l.). During the harvest, potato wart disease appeared in parts of the field. Diseased tubers were collected, and the lengths of the lesions were measured. The percentage of wart infection was calculated as the PLL: the maximum tumor diameters divided by the diameters of transverse sections of tubers multiplied by 100. A total of 24 tubers were classified into eight groups (disease stages DS0–DS7) according to the PLL. The PLL was further used to quantify the area under the disease progress curve, which was calculated with the trapezoidal integration method (Silva et al., 2019). Data were analyzed by ANOVA, with each data point representing the average of three replicates.

### RNA Isolation and Transcriptome Sequencing

Tissue protruding from the surface of the tuber was trimmed off, and the tuber flesh in the lesion was dug out, immediately frozen in liquid nitrogen, and stored at −80°C. Total RNA was isolated from infected and control samples with TRIzol reagent (Thermo Fisher Scientific, Waltham, MA, United States) according to the manufacturer’s recommendations. Briefly, tissue from each sample was ground in TRIzol reagent with four ceramic beads with a tissue homogenizer (MP Biomedicals, Solon, OH, United States) after separation with chloroform, RNA precipitation with isopropanol, and washing of the RNA pellet with 75% ethanol. The air-dried RNA samples were dissolved in water treated with DEPC. The quantity of extracted RNA was detected with a spectrophotometer (NanoDrop™, Thermo Scientific), and the quality was measured by gel electrophoresis.

To construct the library of RNA sequences, we used a NEBNext Ultra RNA Library Prep Kit for an Illumina platform (New England Biolabs, Ipswich, MA, United States) according to the manufacturer’s instructions. We generated the RNA-seq libraries using the NEBNext UltraTM RNA Library Prep Kit for Illumina (NEB) according to the manufacturer’s instructions. Each library was indexed using NEBNext Multiplex Oligos for Illumina (Index Primers Set 1). The 24 libraries were paired-end sequenced by Illumina’s HiSeq 2500 system (Illumina, United States). All raw sequence data as fastq files from the 24 libraries were deposited in the National Center for Biotechnology Information (NCBI)’s sequence read archive (SRA) database with respective accession numbers under BioProject accession number PRJNA803348.

### RNA-Seq Data Processing and Differential Gene Expression

We assessed the quality of the raw reads with Cutadapt v1.10 (Martin, 2011). Reads were aligned with the potato reference genome (group Phureja DM v4.04; Diambra, 2011) with Gmap/Gsnap (Wu et al., 2016) and Samtools (Li et al., 2009). Normalized gene expression levels were calculated with Cufflinks v2.2.1 (Trapnell et al., 2012) and reported as FPKM. The extremely low expressed genes with an average FPKM value below four were excluded from analysis. To understand variability among biological replicates, pearson correlation coefficients were calculated for the log2 transformed FPKM values of the genes expressed in both replicates at a particular disease stage.

Based on the alignments, the read counts were used to perform differential gene expression analysis with DESeq2 (Love et al., 2014). Briefly, we compared infected samples (DS1–DS7) to controls with no symptoms of *S. endobioticum* infection (DS0) using a generalized linear model to obtain log2 fold change differences and corresponding value of *p* for individual transcripts. Also, we compared the samples in the latter disease stage with the previous disease stage (DSn vs. DSn-1). To define DEGs, we subjected the DESeq2 output to the Benjamini–Hochberg method (Ferreira, 2007) for multiple hypothesis testing and filtered it to retain genes with |log2FC| ≥ 1, false discovery rate < 0.05.

To determine the functional annotation of DEGs, the Database for Annotation, Visualization, and Integrated Discovery (DAVID, 2021 Update) was used for Gene ontology (GO) terms annotation and KEGG pathways analysis, with enrichment score >1, and value of *p* < 0.05 defined as significant.

### GCN Analysis

The normalized read counts obtained from DESeq2 were used to construct GCNs with the weighted gene coexpression network analysis (WGCNA) package in R (Langfelder and Horvath, 2008). A one-step network building and module detection approach was used to build a GCN. WGCNA defines a network by connecting all variables in the data set, then detects modules with highly similar expression patterns. First an unsigned topological overlap matrix (TOM) is created to identify a threshold value for module detection. The network construction parameters included a threshold power of nine, a minimum module size equal to 30, and a branch merge cut height of 0.25. The resulting coexpression modules were visualized in Cytoscape v3.6.1 (Shannon et al., 2003). For each module, the top 10 percent of the nodes ranked by connectivity were recognized as hub nodes (genes). To identify the functions of each coexpression module, we performed GO and KEGG enrichment analysis using DAVID. Each gene set was compared against the fully expressed genes as background. Raw value of *p* were corrected for multiple tests and false discovery rate < 0.05 were identified as significantly enriched.

### Confirmation and Quantification of the DEGs

For the confirmation and quantification of the DEGs obtained by the NGS analysis, we conducted RT-qPCR, respectively, for seven DEGs belonging to hub genes in GCN analysis with specific primers (Supplementary Table S1). Total RNA was treated with DNase I (Takara Bio, Shiga, Japan) and reverse-transcribed using the PrimeScript Reverse Transcription reagent Kit (Takara Bio, Shiga, Japan) following the manufacturer’s instructions. RT-qPCR reactions were performed in a CFX Connect Real-Time PCR system (Bio-Rad Laboratories, Hercules, CA, United States), using TB Green Premix Ex Taq II Kit (Takara Bio Inc., Shiga, Japan). TB green RT-qPCR amplification was carried out in 20 μl reaction volumes that contained 2 μl of cDNA template, 10 μl of TB Green Premix Ex Taq II and 0.4 μM each of the forward and reverse primers, respectively, with the following conditions: initial denaturation at 95°C for 2 min, followed by 40 cycles at 95°C for 15 s and 60°C for 30 s. The relative quantification of four DEGs in different DS was carried out using the protocol described above and normalized with internal controls GADPH according to the 2^−ΔΔCT^ method (Livak and Schmittgen, 2001). Three biological replicates were run for each RT-qPCR reaction. Gene expression data were subjected to one-way ANOVA, followed by the Tukey’s HSD *post hoc* test (*p* ≤ 0.05).

## Results

### Assessment of Disease in Potato Tubers Exposed to *Synchytrium endobioticum* Infection

**Figure 1:**
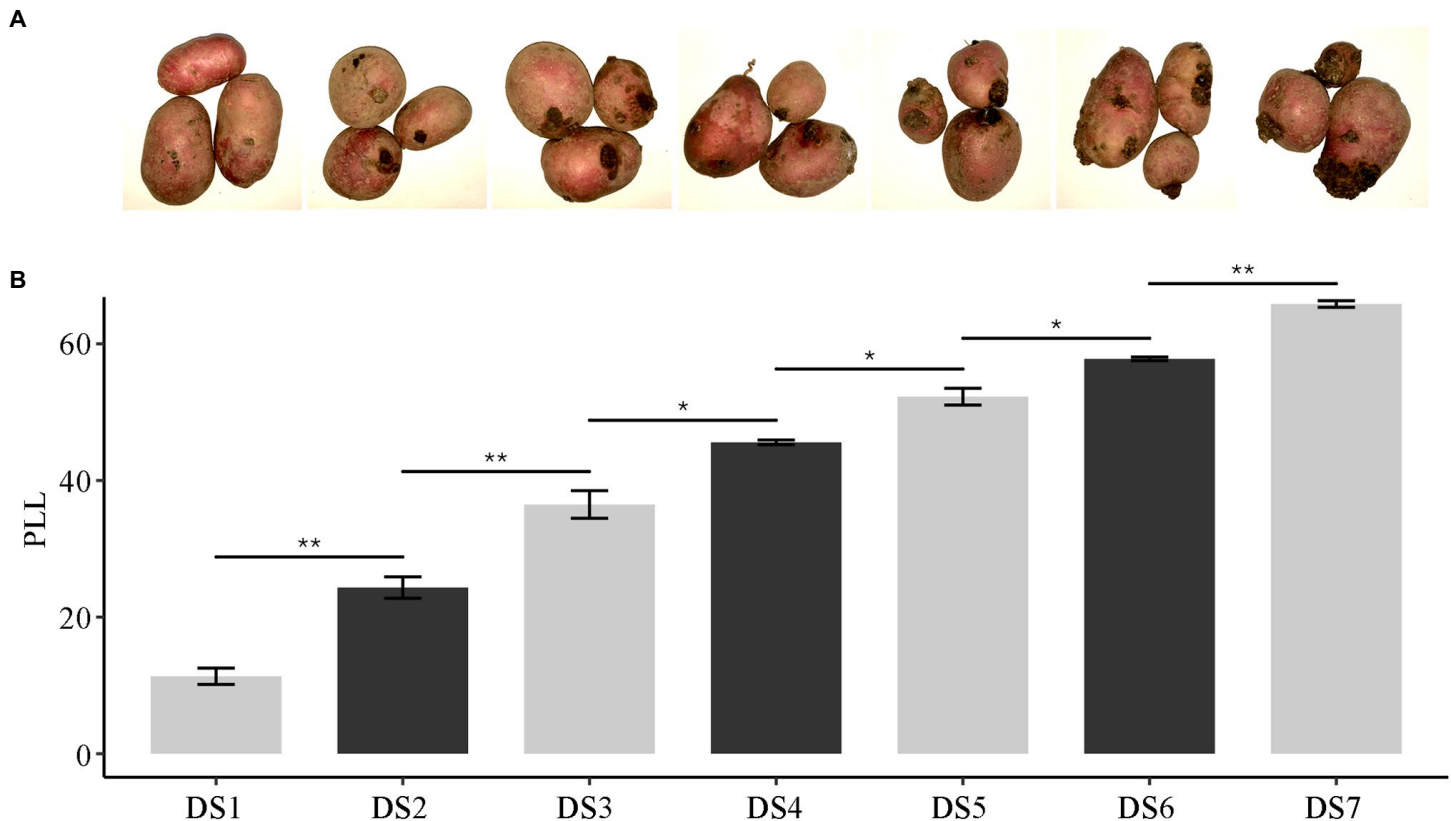
The severity of potato wart disease in tubers infected by *Synchytrium endobioticum*. **(A)** Photographs showing the progression of disease over seven stages. **(B)** The severity of disease expressed as the percent lesion length (PLL). * means *p* < 0.05 and ** means *p* < 0.01, assessed by one-way ANOVA.

To correlate observable symptoms of disease caused by the pathogen with gene expression in host responses, we monitored *S. endobioticum* infections of varying severity in tuber warts of the susceptible potato cv. Qingshu 9. Samples were classified into disease stages DS0–DS7 based on maximum tumor diameters divided by the diameters of transverse sections of tubers, also called the percent lesion length (PLL). ANOVA revealed mutually exclusive stages (Figure 1). DS0 represented the controls, with no symptoms; increasing stages revealed the development of the wart lesions until the tumor size exceeded 60%. Spots of *S. endobioticum* infection were first visible by DS1, on which no tumors yet appeared, corresponding to zoospore encystment and initial penetration through the stomata into the host. The first visible wart was apparent by DS2; warts increased in intensity and became slightly necrotic at the center by DS6. The mean severity of the entire sample was 47.05%, which fell just below the middle stage (DS4) on the scale. The area under the disease progress curve (AUDPC), assessed by one-way ANOVA, was lowest for DS1 at 17.84 and highest for DS7 at 190.83 and also demonstrated that the progression of the disease was in line with the scale.

### Global Transcriptome Patterns Related to *Synchytrium endobioticum* Infection

**Figure 2:**
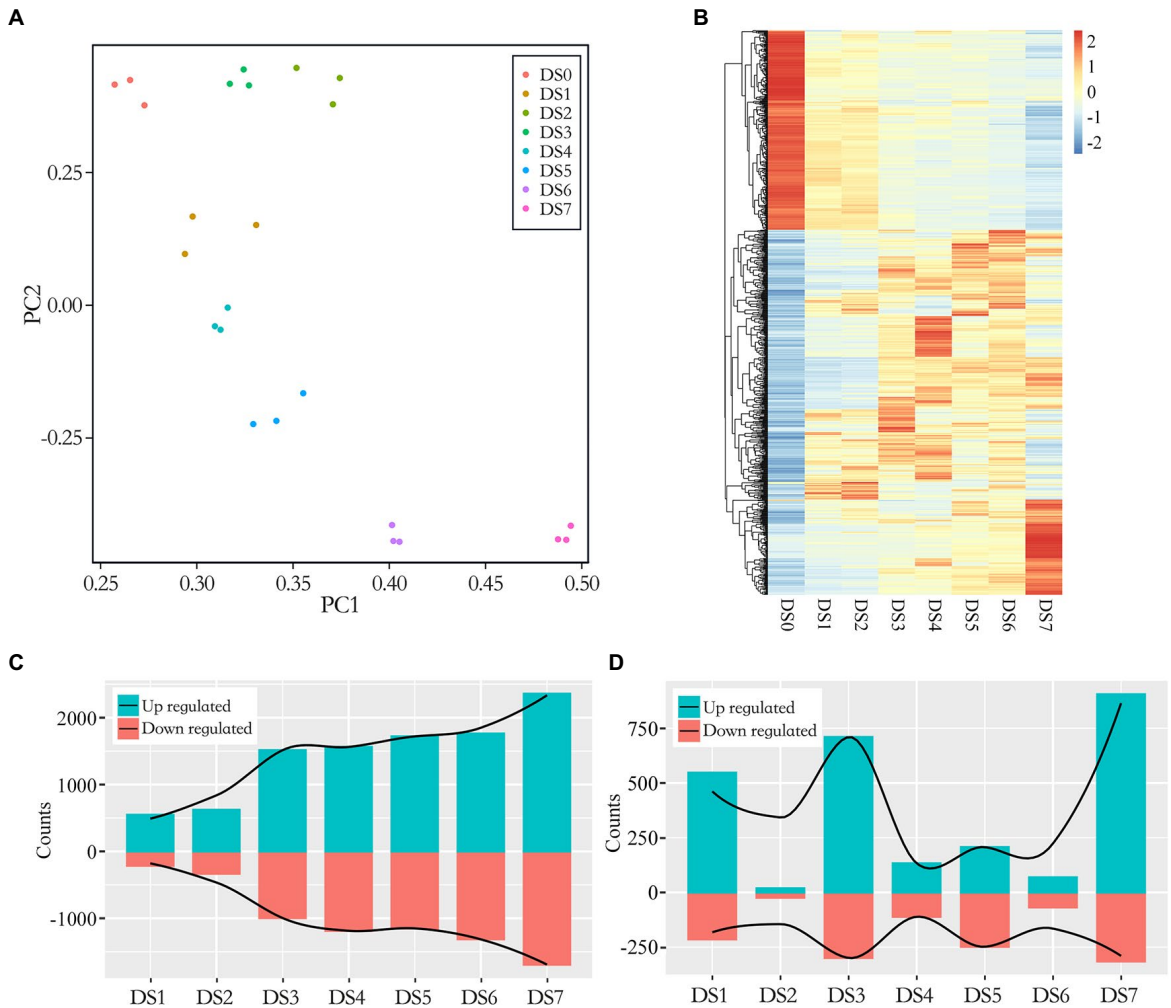
Multiple plots of differentially expressed genes (DEGs) in seven disease stages. **(A)** Principal component analysis (PCA) of log2-transformed normalized gene expression in control and infected samples. **(B)** Clustered heat map of DEGs. Expression was calculated as the log2 fold change (LFC) of the FPKM value. The *x*-axis shows the disease stage, and the *y*-axis shows a dendrogram of samples at each individual stage, indicated by color; genes are represented by individual lines on the *y*-axis. **(C,D)** Bar graphs of the number of up- and down-regulated genes between the control and infected samples (**C**, DSn vs. DS0) as well as between the latter disease stage with the previous disease stage (**D**, DSn vs. DSn-1).

We performed dual mRNA-Seq profiling of wart sites in infected tubers. Three biological replicates were sequenced for each severity level, yielding approximately 1.72 billion raw reads of 24 samples, with 32.11–42.63 million reads per sample. The libraries were constructed from infected potato tubers, and therefore the reads represented transcripts from the host (potato) and the pathogen (*S. endobioticum*). Raw reads were quality-filtered and aligned against the latest potato reference genome (with mapping rates ranging from 32.90% to 89.58%) as well as the *S. endobioticum* reference genome (with mapping rates ranging from 1.42% to 30.26%; Supplementary Table S2). In the early stages of infection, nearly all reads were of host origin. Susceptible interaction in advanced stages of infection led to an increase in pathogen biomass as well as pathogen transcripts in the transcriptome pools. To study the consistency in sampling and biological replicates, we used principal component analysis (PCA) to cluster samples. The results showed the co-localization of biological replicates at each stage (Figure 2A), indicating severity-specific clustering in the wart infection transcriptomes explained by differences in gene expression patterns.

Over all infected samples, a total of 5,052 potato genes were differentially expressed compared to the control samples. A heat map of genes differentially expressed between the control and infected samples is shown in Figure 2B. The dendogram on the x axis showed that genes in DS0 clustered together, all infected sample clusters tended to group together by severity of disease. We compared the gene expression patterns and identified up- and down-regulated DEGs between the infected and control samples (Figure 2C). In general, 15.06%–80.17% out of DEGs were identified in different disease stages and the number of DEGs tended to increase along with the development of the disease. The proportion of DEGs in the early disease stages as DS1 and DS2 were below 20% while increased substantially after DS3 which exceeding 50% as the severity of disease increased (50.68%–60.87% from DS3 to DS6) and jumped to 80% in the last stage DS7. It was suggested a correlation between gene expression and the severity of infection. To elaborate on this step-up trend, we compared the DEG numbers in the latter disease stage with the previous disease stage (DSn vs. DSn-1). As shown in Figure 2D, the DS1, DS3 and DS7 showed substantially increase of DEGs in comparison of the previous stage, representing the early, intermediate and advanced stages of disease development.

**Figure 3:**
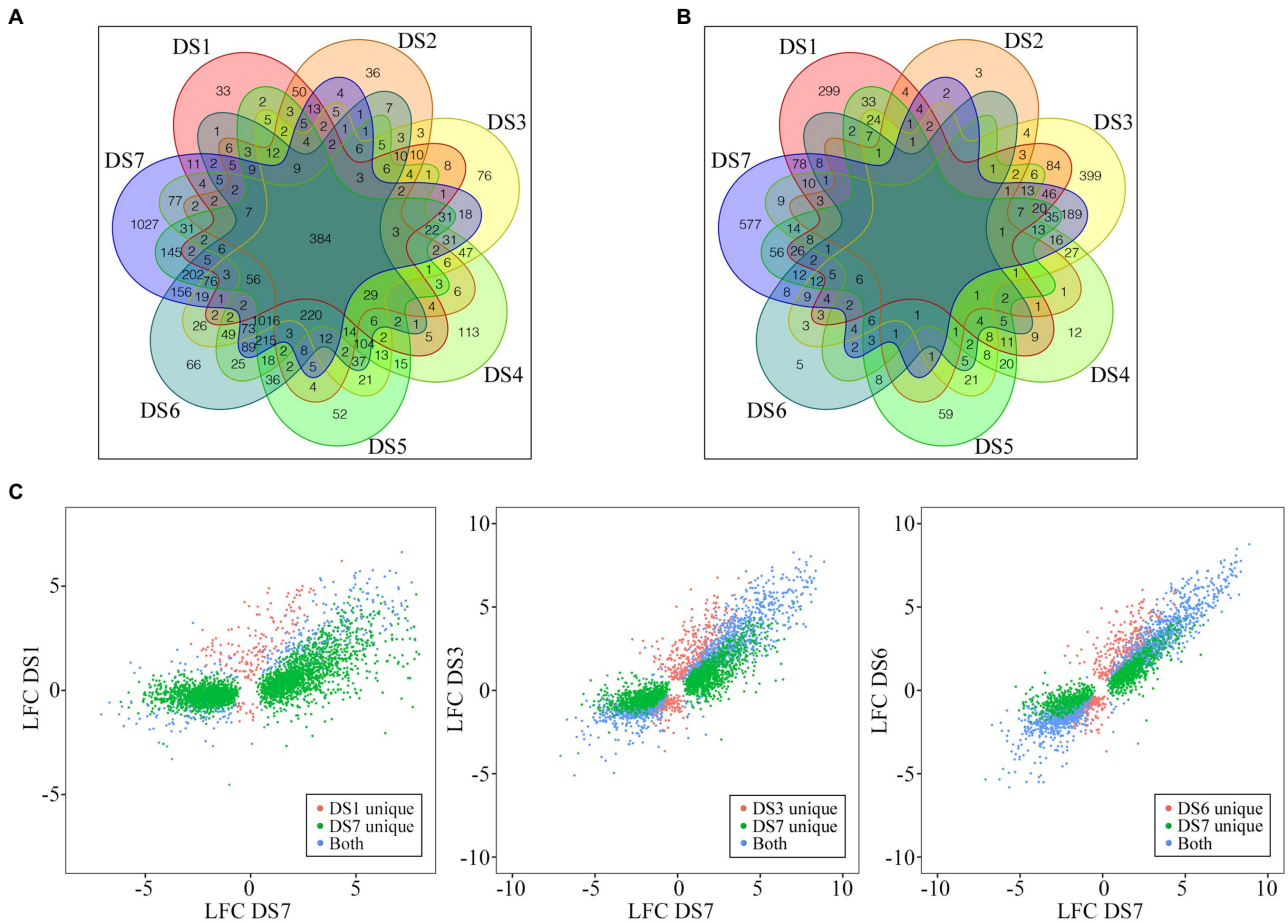
Distribution of the common and unique DEGs among different disease stages. **(A,B)** Evaluation of similarities of DEGs between the control and infected samples (**A**, DSn vs. DS0) as well as between the latter disease stage with the previous disease stage (**B**, DSn vs. DSn-1) using Venn diagrams. **(C)** Pairwise comparisons of unique and conserved genes pair-wisely. Comparisons of DEGs for DS1 vs. DS7, DS3 vs. DS7, and DS6 vs. DS7 are shown.

A Venn diagram of DEGs showed that only 384 DEGs were common to all seven stages of infection. More important, DS7 had the most unique DEGs (1,027), and the number of unique DEGs decreased as the severity of disease decreased, to only 43 unique DEGs in DS1 (Figure 3A). Although DS3 and DS4 had more unique DEGs than DS5 and DS6, the difference was not significant. The differential number of unique DEGs demonstrated that different sets of response genes were likely activated in severely infected samples. As shown in Figure 3B, in the comparison of DSn and DSn-1, the stage with substantial increase of DEG numbers also had a large number of unique DEGs, in which DS1, DS3 and DS7 had 299, 399 and 577 unique DEGs, respectively. Comparisons of the DEGs between early (DS1) and advanced (DS7) stages showed an absolutely higher number of unique DEGs in DS7. However, when compared with the intermediate stage DS3, the DS7-unique DEGs decreased and an increase number of DEGs were common in both stages. Comparison of two adjacent stages (DS6 vs. DS7) showed that most of the DEGs were common, which suggests that several different genes were involved in the host response to pathogen along with the development of the disease (Figure 3C).

Also, the results of pathogen mapping demonstrated the gene expression pattern in *S. endobioticum* during infection. Compared with DS0, the mapping rate of which was 1.46% in average, the vast majority of pathogen genes (6,398 out of 8,031) expressed differentially in the infected samples. Different from potato DEG patterns, the number of pathogen-DEGs increase in DS1 sharply to 4,573 and stabilize within the range between 4,890 and 5,485 in the subsequent disease stages (Supplementary Figure S1). The most interesting is that the number of upregulated DEGs was 5.2–5.6 times the number of downregulated DEGs, demonstrated that the pathogen gene expression was active rather than suppressed in the process of attacking the host. In addition, few unique DEGs in each stage were identified as 3,223 DEGs were common to all infected stages (Supplementary Figure S2).

### System-Level Functional Analysis in Response to *Synchytrium endobioticum* Infection

**Figure 4:**
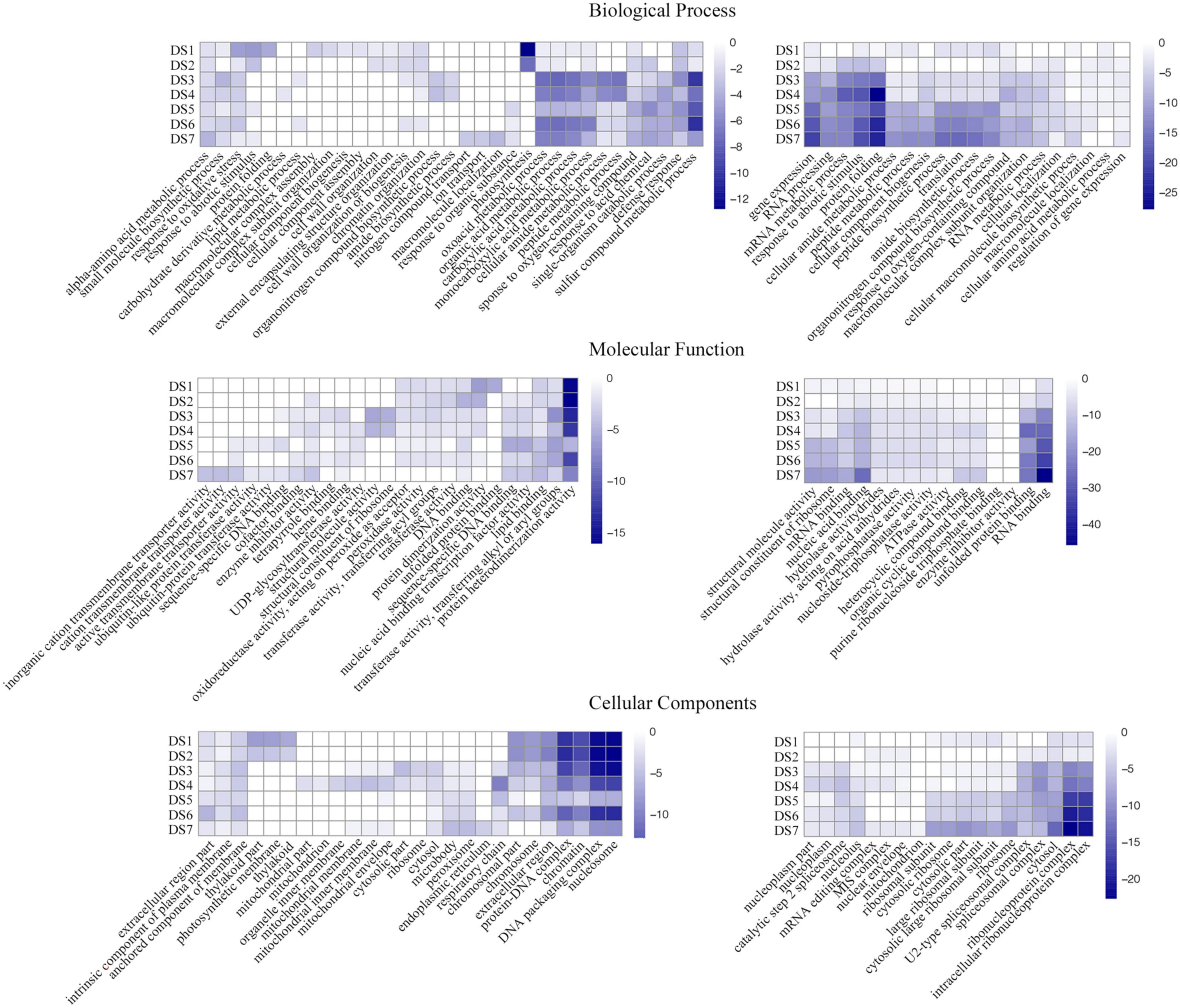
Gene ontology (GO)-term correlation plots comparing probabilities of GO-term enrichments among different disease stages. Heatmaps in left and right represented the GO terms with the up- and down-regulated DEGs between each infected samples and the control, respectively. Color key scale indicates the minus log10 value of *p* GO term significance.

Analyses of GO term and KEGG pathway enrichment were used to identify differential responses to potato wart at system-level functional pathways. To gain further insights about the pathway functions, we separated the up- and down-regulated DEGs between each infected samples and the control, as well as stage-specific DEGs between the latter and the previous disease stages. The top 10 enriched GO terms, including biological process, molecular function and cellular components were shown in Figure 4; Supplementary Tables S3 and S4. Biological process specific to defense response (GO:0006952) were upregulated and organonitrogen compound biosynthetic process (GO:1901566) were downregulated in all infected stages. Photosynthesis (GO:0015979) and cell wall organization (GO:0071555) were only upregulated during the early stage and peptide metabolic process (GO:0006518) were upregulated during the intermediate and advanced stages. In the last stage DS7, nitrogen compound transport (GO:0071705) and ion transport (GO:0006811) were specifically enriched. As to the molecular function, DNA binding (GO:0003677), transferase activity (GO:0016765), lipid binding (GO:0008289) and protein heterodimerization activity (GO:0046982) were shown upregulation whereas RNA binding (GO:0003723) and nucleic acid binding (GO:1901363) were shown downregulation in all infected stages. As the disease progresses, functions related to active transmembrane transporter activity (GO:0022804) and ubiquitin-protein transferase activity (GO:0019787) were induced at DS5-7.

A greater number of significantly (*p* < 0.05) enriched KEGG pathways were identified at each disease stages (Supplementary Tables S5 and S6). Up-regulated DEGs displayed a total of 84 enriched pathways and down-regulated DEGs enriched in 61 pathways. Plant-pathogen interaction (sot04626), fatty acid elongation (sot00062), phenylpropanoid biosynthesis (sot00940), phenylalanine metabolism (sot00360) and glutathione metabolism (sot00480) were associated with the up-regulated genes in all seven infected stages. However, some pathways enriched with up-regulated genes were specifically enriched at different disease stages. Early stages DS1 and DS2 showed significant enrichment of pathways related to photosynthesis (sot00195) and porphyrin metabolism (sot00860) while pathways related to MAPK signaling pathway (sot04016) was induced since DS3. Interestingly, the last and most advance stage (DS7) had specific gene enrichments for pathways related to phagosome (sot04145) and metabolism of unsaturated fatty acids including biosynthesis of unsaturated fatty acids (sot01040) and α-linolenic acid metabolism (sot00592). In contrast, mRNA surveillance pathway (sot03015) and spliceosome (sot03040) showed downregulation in all seven infected stages. Pathways specific to pantothenate and CoA biosynthesis (sot00770) and ubiquitin mediated proteolysis (sot04120) were downregulated during the intermediate and advanced stages of disease development.

### Gene Coexpression Patterns Associated With *Synchytrium endobioticum* Infection

**Figure 5:**
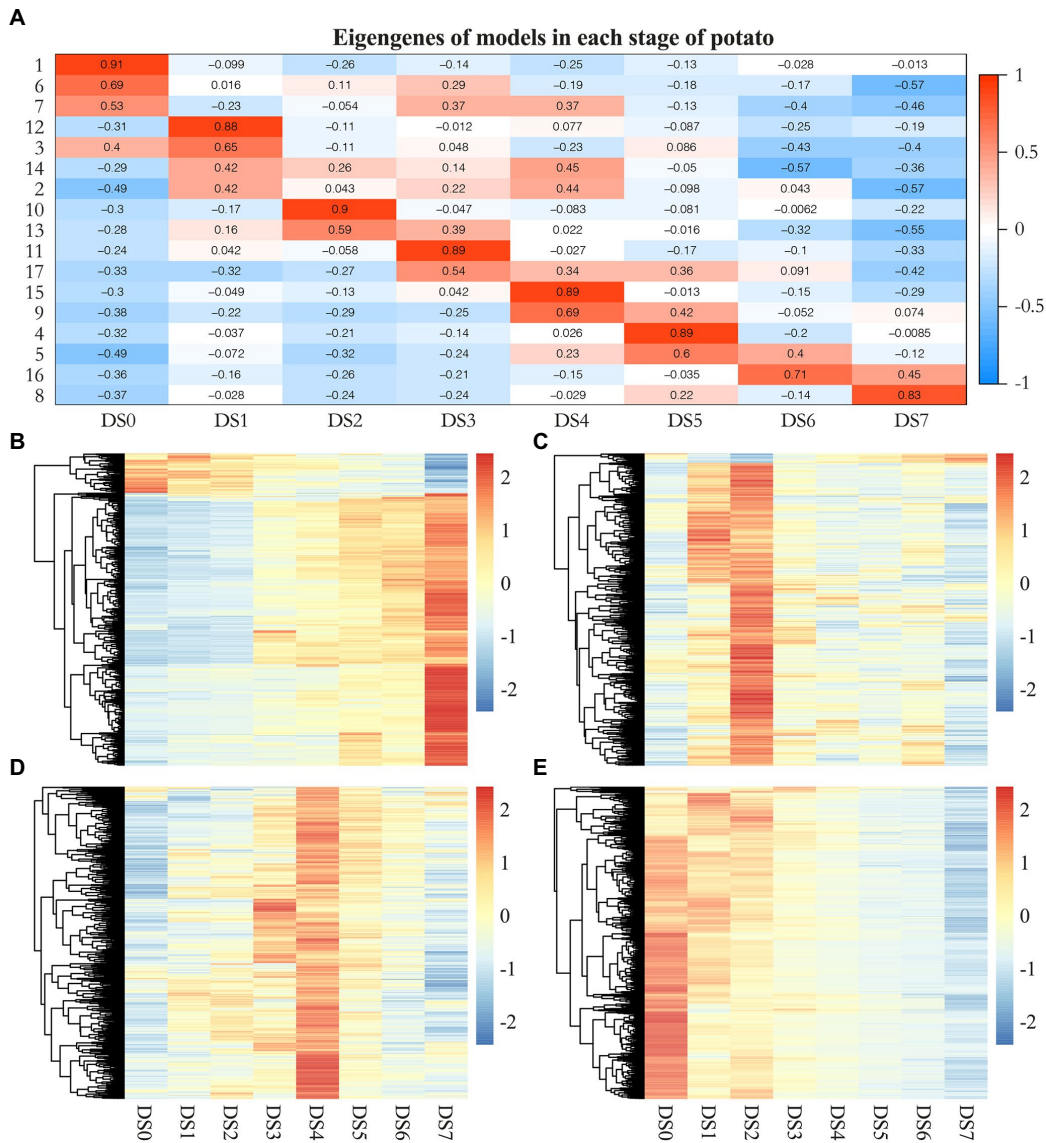
Weighted gene coexpression network analysis (WGCNA) of genes differentially expressed following *S. endobioticum* infection in potato. **(A)** Heat map of eigengenes of each module correlated with the different infection stages. **(B–E)** Gene expression patterns in four coexpression modules that specifically showed enrichment for response to biotic stimuli.

We categorized genes differentially expressed between control samples and samples with varying levels of disease severity into distinct gene networks and used WGCNA to explore gene expression patterns and regulatory network responses to potato wart disease. A total of 4,621 highly correlated genes formed modules in which all members were more highly correlated with each other than they were with genes outside the module. WGCNA identified 17 distinct gene modules that contained between 25 and 813 genes. These highly interconnected GCN modules (subnetworks) were more likely to share common biological function or regulatory mechanisms. To visualize the module traits with respect to the progression of infection, we correlated eigengenes of each module with the different infection stages. As shown in Figure 5A, although a few modules included genes that shared broader coexpression patterns across multiple stages of infection, most modules differed significantly at certain stages of the stress response. For example, modules 1 and 6 were representative of genes with correlated coexpression at control, which enriched the largest number of genes highly expressed in uninfected samples and coordinately downregulated their expression after infection. Modules 12 and 10 were representative of genes highly coexpressed in the early stage (DS1 and 2) that might be responsible for inducing defense signaling in the host. The gene expression in modules 4, 11, and 15 showed peak upregulation in DS3, DS4, and DS5, respectively, corresponding to the middle stages of infection. Moreover, modules 16 and 8 had peak expression in DS6 and DS7 alone, which implicated them in serious susceptible responses in the late stages of infection. Gene expression patterns in 17 coexpression modules were shown in Figures 5B–E; Supplementary Figure S3.

We further performed GO and KEGG enrichment analysis to characterize functional classes and pathways associated with individual coexpression modules (Supplementary Table S7). The results of GO enrichment showed system-level functional activity initiated by pathogen infection. Five coexpression modules showed particular overrepresentation for the stress response. Module 1 and 8 had prominent enrichment of response to abiotic stimulus (GO:0009628). In addision, Module 8 showed enrichment for defense response (GO:0006952) and a series of GO terms related to gene positive regulations; Module 9 overrepresented GO terms related to cell wall organization or biogenesis (GO:0071554), response to oxidative stress (GO:0006979) as well as several negative regulations of biosynthetic process. Module 10 had a number of enriched GO terms mainly involved in regulation process and Module 17 showed significant enrichment of defense response to biotic stimulus (GO:0009607). In the results of KEGG enrichment, five modules (M3, M5, M6, M7, and M14) did not show significant enrichment for any KEGG pathway. Some pathways were specifically identified in a single module. Among them, Module 1 had prominent enrichment of spliceosome (sot03040), mRNA surveillance pathway (sot03015) and nucleocytoplasmic transport (sot03013). Module 8 showed enrichment for plant hormone signal transduction (sot04075) and MAPK signaling pathway (sot04016). Biosynthesis of various alkaloids (sot00996) was specifically enriched in Module 10. In contrast, several functional pathways were observed in more than one cluster. For example, both module 4 and 17 showed enrichment for terpenoid backbone biosynthesis (sot00900) and pyruvate metabolism (sot00620); module 2 and 11 revealed the enrichment of ubiquitin mediated proteolysis (sot04120).

Based on the WGCNA results for these notable modules, we identified hub genes that played crucial roles during infection and visualized the functional networks shown in Supplementary Figure S4. To validate the expression pattern of identified hub genes in the control and samples of seven infected stages, quantitative RT-qPCR were performed on the hub genes with highest connectivity in Module 1, 8, 9, and 10, respectively. The results of RT-qPCR showed the similar expression pattern of these four genes with RNA-Seq results and pearson correlation coefficients estimated from RNA-Seq and RT-qPCR ranged from 0.778 to 0.956 (Supplementary Figure S5). As shown in Figure 5B, genes in module 8 were significantly upregulated compared to the uninfected controls. This set of 48 hub genes included a large suite of genes implicated in the pathogen response, including class II chitinase (PGSC0003DMG400001528), Kunitz-type protease inhibitor (PGSC0003DMG400015267), β-ketoacyl-CoA synthase family protein (PGSC0003DMG400007373), nodulin (PGSC0003DMG400015263), CBL-interacting protein kinase (PGSC0003DMG400011106), late embryogenesis abundant protein 5 (PGSC0003DMG400017936). The common characteristics of these gene expression patterns were low expression in the controls and increased gene expression to thousands across multiple stages of infection and to peak expression in the last stage (DS7), which suggests an active attempt by the pathogen to alter host defense responses. Similarly, genes in module 10 were significantly upregulated compared to the controls, having the highest expression in the early stage (DS2) but becoming increasingly downregulated as the infection got worse (Figure 5C). The 15 hub genes in this module, which were responsible for inducing defense signaling in the early stages of infection, were ethylene response factor 5 (PGSC0003DMG400040046), Avr9/*Cf*-9 rapidly elicited protein (PGSC0003DMG400012296) and kinase interacting protein (PGSC0003DMG400027049). In module 9, we identified 35 hub genes related to plant defense (Figure 5D), in which ring finger protein (PGSC0003DMG400030821) had the highest connectivity of 155, followed by UDP-glucosyltransferase (PGSC0003DMG400024618), Class III peroxidase (PGSC0003DMG400000511), NADH dehydrogenase (PGSC0003DMG400005271), extensin (PGSC0003DMG400000776), lipid binding protein (PGSC0003DMG401023630). They were significantly upregulated in the early stages of infection, reaching peak expression in the middle stage (DS4) of the defense response. Given the fact that the majority of DEGs were upregulated during defense responses, module 1 included genes highly expressed in the controls but increasingly downregulated in infected samples (Figure 5E). Among 81 hub genes in this module, gene encoding histone acetyltransferase (PGSC0003DMG401015202) had the highest connectivity of 701. Several other transferase encoding genes such as methyltransferase (PGSC0003DMG400024216), glycosyltransferase (PGSC0003DMG400020103), acetylglucosaminyltransferase (PGSC0003DMG400008939) as well as RNA binding protein such as SGRP-1 protein (PGSC0003DMG400033903), ran binding protein (PGSC0003DMG400020543), RNA-binding region RNP-1 (PGSC0003DMG400029841) showed either high connectivity in this module.

## Discussion

A significant difference was elaborated for gene expression under disease progression when infected with pathogen of potato wart, demonstrating unique biological processes and molecular mechanisms leading to susceptible responses. It is of fundamental importance to uncover gene changes in susceptible potato cultivars following infection, which will help to exploit systemic response, especially susceptible responses to pathogen. In the biological process enrichment, DEGs up-regulated throughout all disease stages were related to defense response, plant-pathogen interaction as well as fatty acid elongation and phenylpropanoid biosynthesis. The process of fatty acid elongation had been reported in responses of plants to abiotic and biotic stresses, which are of primary importance for many interactions of the plant with its surrounding environment caused by pathogen or insect attacked and serves as a protection against pathogen infection to sustain post-injury survival (Batsale et al., 2021). On the other side, long-chain fatty acids such as sphingolipid was characterized as a positive regulator in biotic response and plant defense against fungi and bacteria pathogens by activating programmed cell death leading to spontaneous necrosis and lesions, also called accelerated cell death phenotype (Michaelson, 2011; Berkey et al., 2012). In plant–pathogen interactions, the phenylpropanoid pathway plays a critical role in plant defense response to fugal pathogen, in which previous studies in lettuce and tomato had been reported gene related to phenylpropanoid biosynthesis induced during the compatible interaction with *Botrytis cinerea* (De Cremer et al., 2013) and *Verticillium dahlia* (Tan et al., 2015).

The GCNs in our study presented distinct modules of genes enriched in the biotic stress response, suggesting a potential transcriptional regulatory network underlying the global response in different stages of infection. In general, pathogen infection should induce the downregulation of metabolic processes to conserve energy for resistance. However, the upregulation of metabolic processes during pathogen infection may trigger signal cascades that lead to host resistance. Module 8, with genes increased expression along with the disease progression, showed enrichment for plant hormone signal transduction and MAPK signaling pathway and involved pathogenesis-related hub genes encoding kunitz-type protease inhibitor and nodulin protein. As far as we know, kunitz-type protease inhibitors, showed high homology with miraculin-like proteins, is involved in the endogenous defense system as it helps regulate and balance protease activity, which plays a pivotal role in maize defense signaling and the regulation of plant cell death associated with the pathogen response involving the interplay of activating triggers and inhibitors (van der Linde et al., 2012). The induction of nodulin proteins in potato–*S. endobioticum* interactions has been associated with improvements in pathogen fitness through control over plant transporters. As reported before, the accumulation of transporters for nutrient uptake represents a shift from the biotrophic phase to the necrotrophic phase in hemibiotrophic pathogens due to the rapid growth of secondary hyphae in the late stages of infection (Zamora-Ballesteros et al., 2021).

Exploration of gene functions in pathways activated in the early stages of infection can provide knowledge about pathogen invasion as well as quick and effective responses among host plants. Module 10 was composed of genes with peak expression in the early stages of infection, and represented groups associated with biological regulation process in functional enrichment. The upregulation of ethylene-responsive transcription factor illustrates the early stress response. In most cases, the induction of ethylene-responsive transcription factor gene expression precedes mRNA accumulation of potential downstream target chitinase genes (Samac et al., 1990; Oñate-Sánchez and Singh, 2002). Although the ethylene-responsive transcription factor gene was upregulated in the early stages of infection and then was quickly tamped down during infection, transcriptional activation cascades may be important for host defense against pathogen attack while inhibited by pathogen with the progression of the infection (Oñate-Sánchez and Singh, 2002). CCR4-associated factor and Avr9/*Cf*-9 rapidly elicited genes had the same expression patterns, which rapidly altered expression in the early stages. CCR4-associated factor is necessary for plant development and defense, and its upregulation might contribute to a thickening of the cell wall through the formation of cellulose, the enlargement of cells, and the fortification of the cell wall through the regulation of downstream peroxidases (Sarowar et al., 2007). Many Avr9/*Cf*-9 rapidly elicited genes encode putative signaling components and regulatory proteins, including protein kinases and transcription factors, and thus may play pivotal roles in the initial development of the defense response (Liang et al., 2009). Specifically, *Cf* genes confer resistance to fungal pathogens by recognizing secreted Avr peptides, and host defense responses are immediately activated when the pathogen is perceived (De Vega et al., 2021). These signaling components could activate downstream R genes such as Rcr-1 and Rcr-2, as could ubiquitylation, which is important for *Cf*-9/Avr9-mediated defense responses (Rowland et al., 2005). Module 1 clearly represented a certain gene expression pattern highly expressed in the controls but increasingly downregulated in infected samples. From the results of functional enrichment in GO and KEGG pathways, we found Module 1 had prominent enrichment of response to abiotic stimulus as well as spliceosome, mRNA surveillance pathway and nucleocytoplasmic transport. And the hub genes in Module 1 contained some known disease resistance genes like transferase encoding genes and RNA binding protein encoding genes. The core hub gene with the highest connectivity was gene encoding histone acetyltransferase. A growing body of evidence suggests epigenetic mechanisms including histone acetylation is pertinent to interactions between hosts and pathogens (Gómez-Díaz et al., 2012). Histone acetyltransferases (HATs) had played a role in the regulation of plant defense responses (Ramirez-Prado et al., 2018). Also, the genes enriched in spliceosome and mRNA surveillance pathway were involved in the main regulatory processes to achieve early effective immunity, through either nonsense-mediated mRNA decay or alternative precursor mRNA splicing variation (Jung et al., 2020). Their downregulation explains their inefficiency in containing infection in earlier stages, which suggests an active virulence mechanism to abrogate host responses associated with stress-induced signaling.

From the results of the functional enrichment, our study showed an activation of basal defense response related genes. It has no doubt that resistant host initiate defense responses in incompatible interactions, while susceptible host can also launch a series of basal defense responses in compatible interactions. Our results demonstrated that there may be an overlap among the susceptible responses of potato to fungal pathogens. As reported before, compatible response of potato inoculated with *Phytophthora infestans* uncovered significant differential expression of many defense- and disease-responsive genes (Restrepo et al., 2005). In fact, several genes and QTLs for potato wart disease resistance have been in potato populations, in which some *Sen* genes were found to be dominant genes giving a qualitative type of resistance (Prodhomme et al., 2020). Referenced to the flanking intervals of these QTLs, a total of 372 different DEGs in our study were located across the major effect QTL regions (Supplementary Table S8). The number of DEGs ranged from 56 for DS1 to 372 for DS7. Analysis of functional annotations of DEGs in potato wart QTLs found candidate gene with previous association with disease resistance. For example, receptor-like protein kinases such as CBL-interacting protein kinase and brassionsteroid insensitive-associated receptor kinase, as well as repeat domain-encoding genes including leucine rich repeat family and F-box/kelch-repeat protein, binding protein encoding genes like MAR-binding protein and ring finger protein were present in QTL regions from all the disease stages. Transcription factors such as WRKY domain, BZIP domain, SCL domain and C3H4 Zinc finger proteins were also among the DEGs in potato wart QTL regions.

Our study of using stages of infection captures the divergent properties observed in the gene expressions of the host and the pathogen by dual RNA-Seq, which was expected to become the gold standard in the studies of host-pathogen interactions (Camilios-Neto et al., 2014). When analyzing the genes expressed by *S. endobioticum*, it was possible to see that the amount of *S. endobioticum* genes detected in the study was highly variable depending on the states of tuber samples, and it ranged from 2,623 to 6,670 genes. The progressive increase of *S. endobioticum* RNA reflected the progression of disease (Supplementary Figure S1A) and the sharply increase of the relative amount of *S. endobioticum* RNA in the DS1 indicated the fungal invasion in large numbers (Supplementary Figure S1B). The pathogenicity-related transcripts included apoplastic effectors and secreted proteins, which were differentially expressed by *S. endobioticum*. As previously reported, many of them were proteins involved in protein degradation and modification (Brilli et al., 2018). Genes involved periplasmic serine protease and cysteine protease up-regulated by *S. endobioticum* might be important for the pathogenicity of pathogen, which is one of the best-characterized virulence factors and is of fundamental importance for invasion and dissemination of the fungus through the stratum corneum of the host (Leng et al., 2009; Bitencourt et al., 2016). Another important category attributed to the pathogen were associated with necrosis or breakdown of host cells, in which genes encoding lectins, 1,3(4)-β-D-glucanases and cellulose-binding proteins were up-regulated by *S. endobioticum* (Hayden et al., 2014). These effectors above were putatively involved in the hydrolysis of antifungal proteins produced by the host as well as the cell wall degradation during colonization.

In summary, infection by the fungal pathogen *S. endobioticum* activates a system-level response in potato tubers. Although further research is needed to determine whether the expression patterns of hub genes in modules are causative biomarkers or simply an effect of root nodulation or pathogen defense pathways, their identification suggests hypotheses to test.

## Data Availability Statement

The datasets presented in this study can be found in online repositories. The names of the repository/repositories and accession number(s) can be found at: https://www.ncbi.nlm.nih.gov/, PRJNA803348.

## Author Contributions

LY, YZ, and XL designed the research. YQ prepared the plant sample. XL and HW performed the transcriptome sequencing, assembly, and annotation. XL, YL, and XT performed the data analysis. LY and YL performed the RT-qPCR experiment. YZ and HW supervised the experiments and analysis. LY and XL wrote the initial draft. YZ, HW, and XT revised the manuscript. All authors contributed to the article and approved the submitted version.

## Funding

This work was supported by the Application Fundamental Project of Science and Technology of Sichuan (no. 2019YJ0546) and the National Natural Science Foundation of China (no. 32060720) to LY.

## Conflict of Interest

The authors declare that the research was conducted in the absence of any commercial or financial relationships that could be construed as a potential conflict of interest.

## Publisher’s Note

All claims expressed in this article are solely those of the authors and do not necessarily represent those of their affiliated organizations, or those of the publisher, the editors and the reviewers. Any product that may be evaluated in this article, or claim that may be made by its manufacturer, is not guaranteed or endorsed by the publisher.

## Supplementary Material

Supplementary Figure S1 Bar graphs of the number of up- and down-regulated pathogen genes between the control and infected samples (**A**, DSn vs. DS0) as well as between the latter disease stage with the previous disease stage (**B**, DSn vs. DSn-1).

Supplementary Figure S2 A Venn diagrams of common and unique differentially expressed genes (DEGs) in different disease stages compared with control sample.

Supplementary Figure S3 Heat maps showing the co-expressed genes in 13 modules.

Supplementary Figure S4 Visualized coexpression network and related modules enriched with infection response genes.

Supplementary Figure S5 RT-qPCR validation of RNA sequencing results. Four hub genes with the highest connectivity in four modules were selected for the confirmation of differentially expressed genes using the same RNA samples that were used for RNA sequencing. Pearson correlation between the gene expression levels measured using RT-qPCR and RNA-Seq was used for validation. The colors in the heat plot represents values for log2 fold change for each sample.

Supplementary Table S1 A set of specific primer pairs for four hub genes with the highest connectivity in four modules in RT-qPCR validation.

Supplementary Table S2 Summary of raw sequence data for dual RNA-Seq and SRA accession number.

Supplementary Table S3 Functional enrichment of the GO terms with the up- and down-regulated DEGs between each infected samples and the control.

Supplementary Table S4 Functional enrichment of the GO terms with the stage-specific DEGs between the latter and previous disease stages.

Supplementary Table S5 Functional enrichment of the KEGG pathways with the up- and down-regulated DEGs between each infected samples and the control.

Supplementary Table S6 Functional enrichment of the KEGG pathways with the stage-specific DEGs between the latter and previous disease stages.

Supplementary Table S7 Functional enrichment of the GO terms and KEGG pathways with the coexpression genes in 17 network modules.

Supplementary Table S8 List of DEGs in the major QTL regions that confer resistance against S. endobioticum infection.
