# Supplementary figures and images for: Integrative Analysis of Genes Involved in the Global Response to Potato Wart Formation
Source: Front Plant Sci. 2022 Jun 29;13:865716. doi: 10.3389/fpls.2022.865716 (PMC9277394; doi:10.3389/fpls.2022.865716)

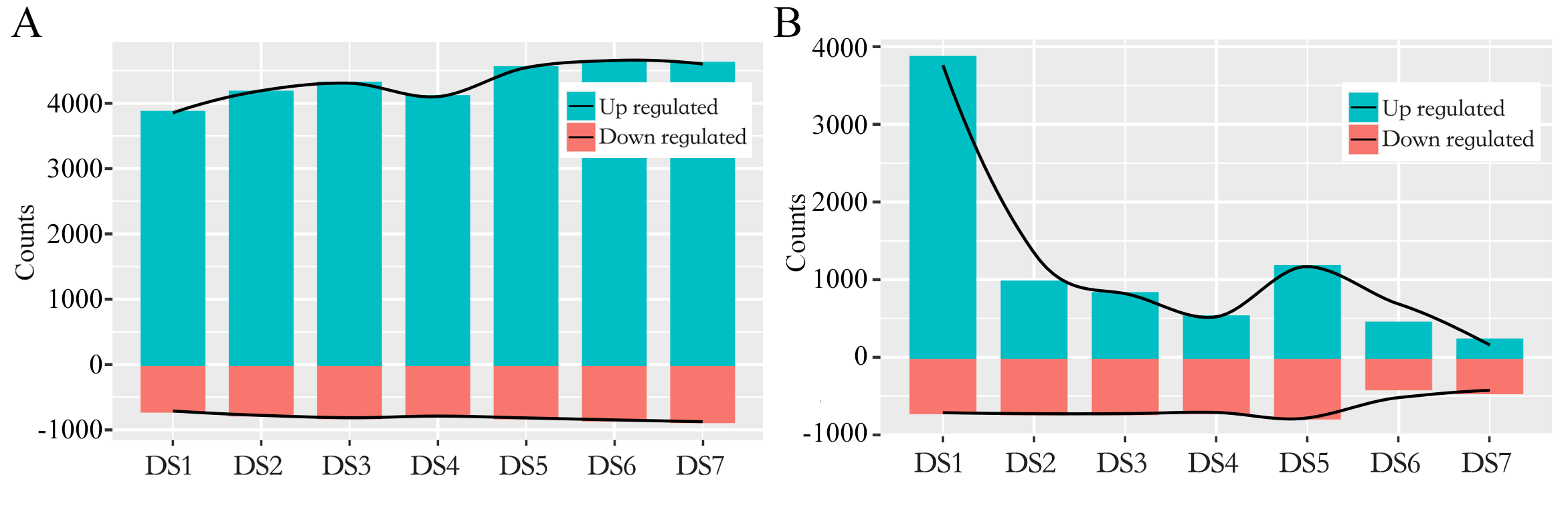

Supplement: Supplementary Figure S1 — Bar graphs of the number of up- and down-regulated pathogen genes between the control and infected samples (A, DSn vs. DS0) as well as between the latter disease stage with the previous disease stage (B, DSn vs. DSn-1). [file Image_1.TIF]

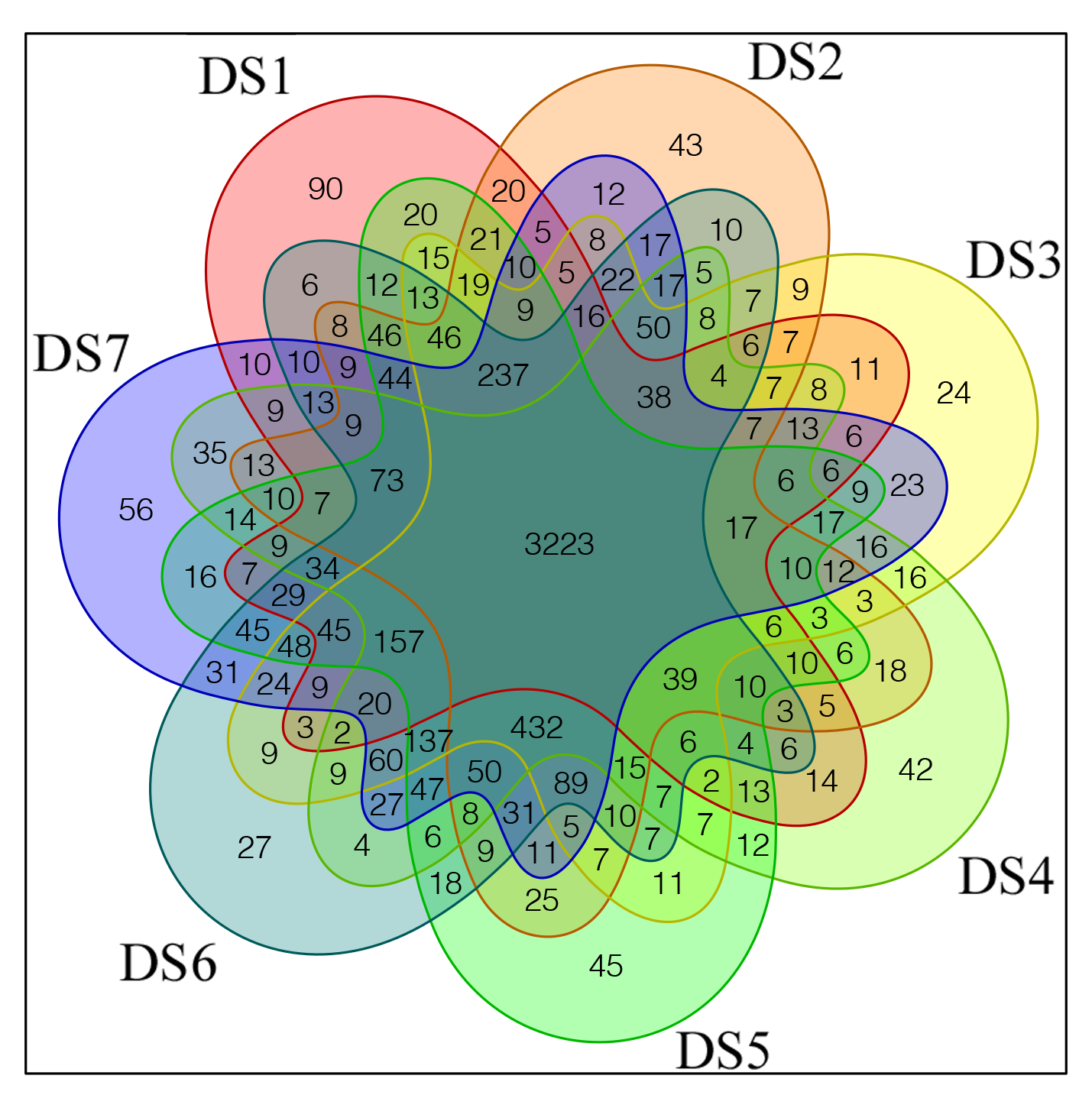

Supplement: Supplementary Figure S2 — A Venn diagrams of common and unique differentially expressed genes (DEGs) in different disease stages compared with control sample. [file Image_2.TIF]

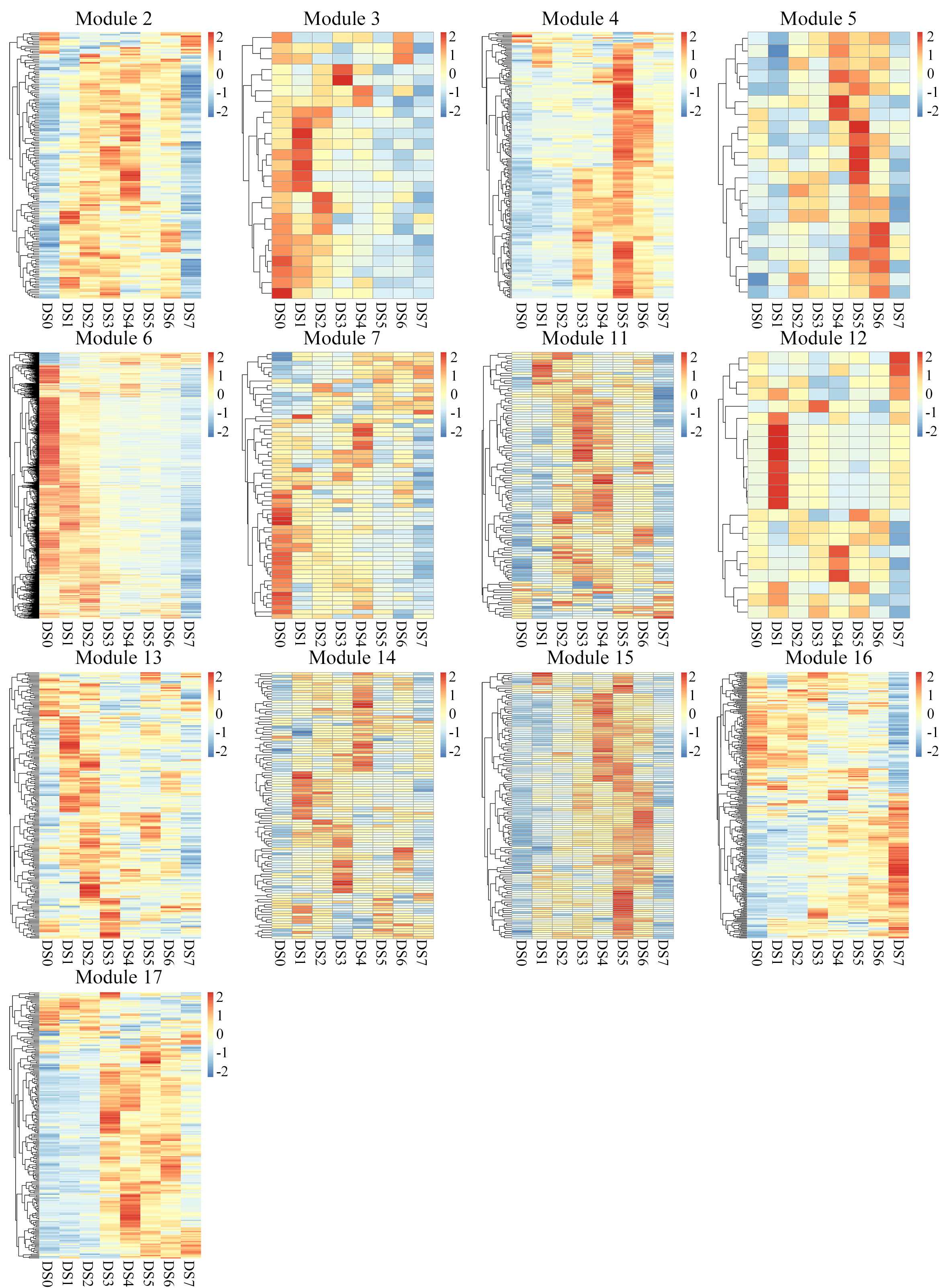

Supplement: Supplementary Figure S3 — Heat maps showing the co-expressed genes in 13 modules. [file Image_3.TIF]

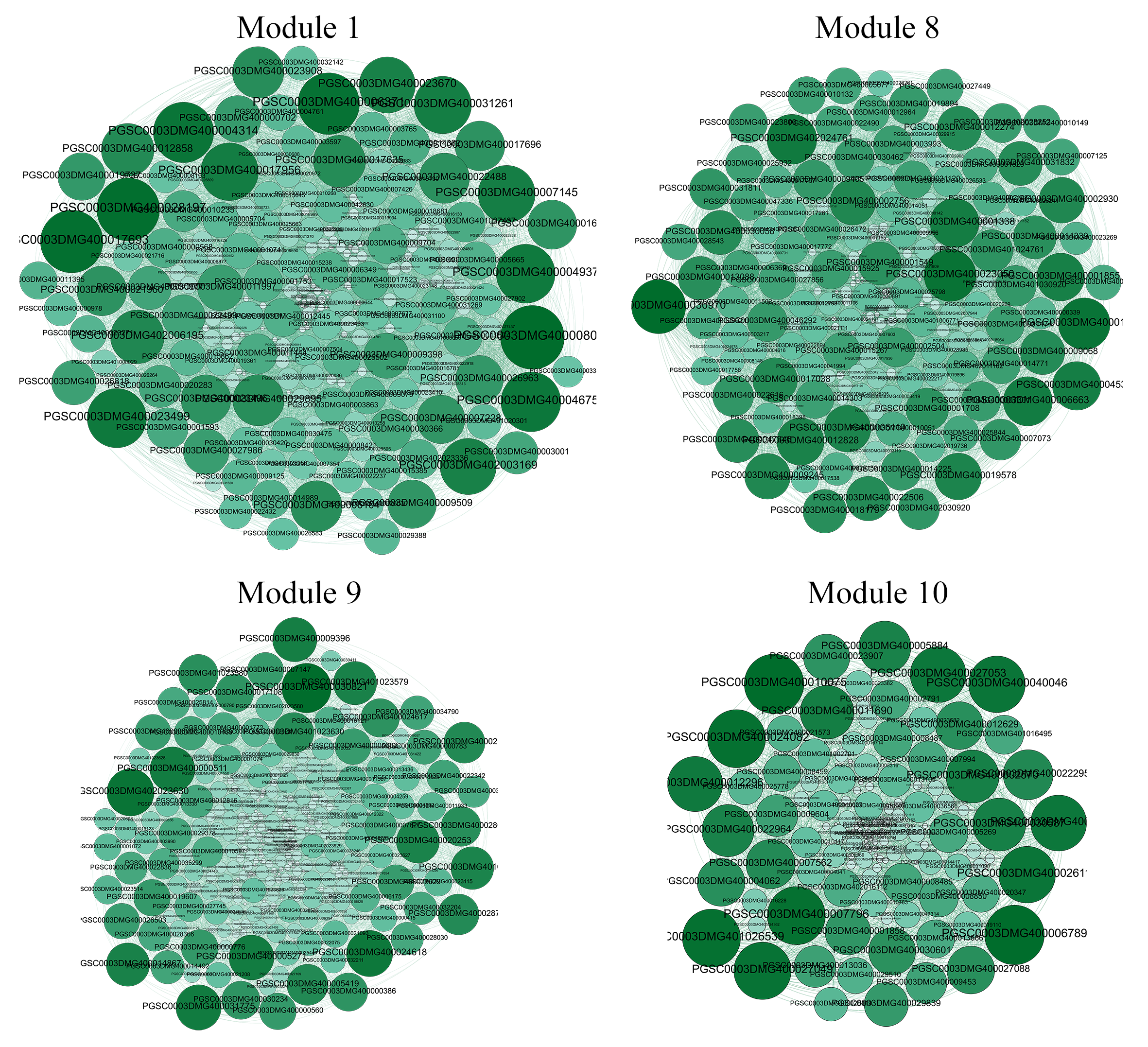

Supplement: Supplementary Figure S4 — Visualized coexpression network and related modules enriched with infection response genes. [file Image_4.TIF]

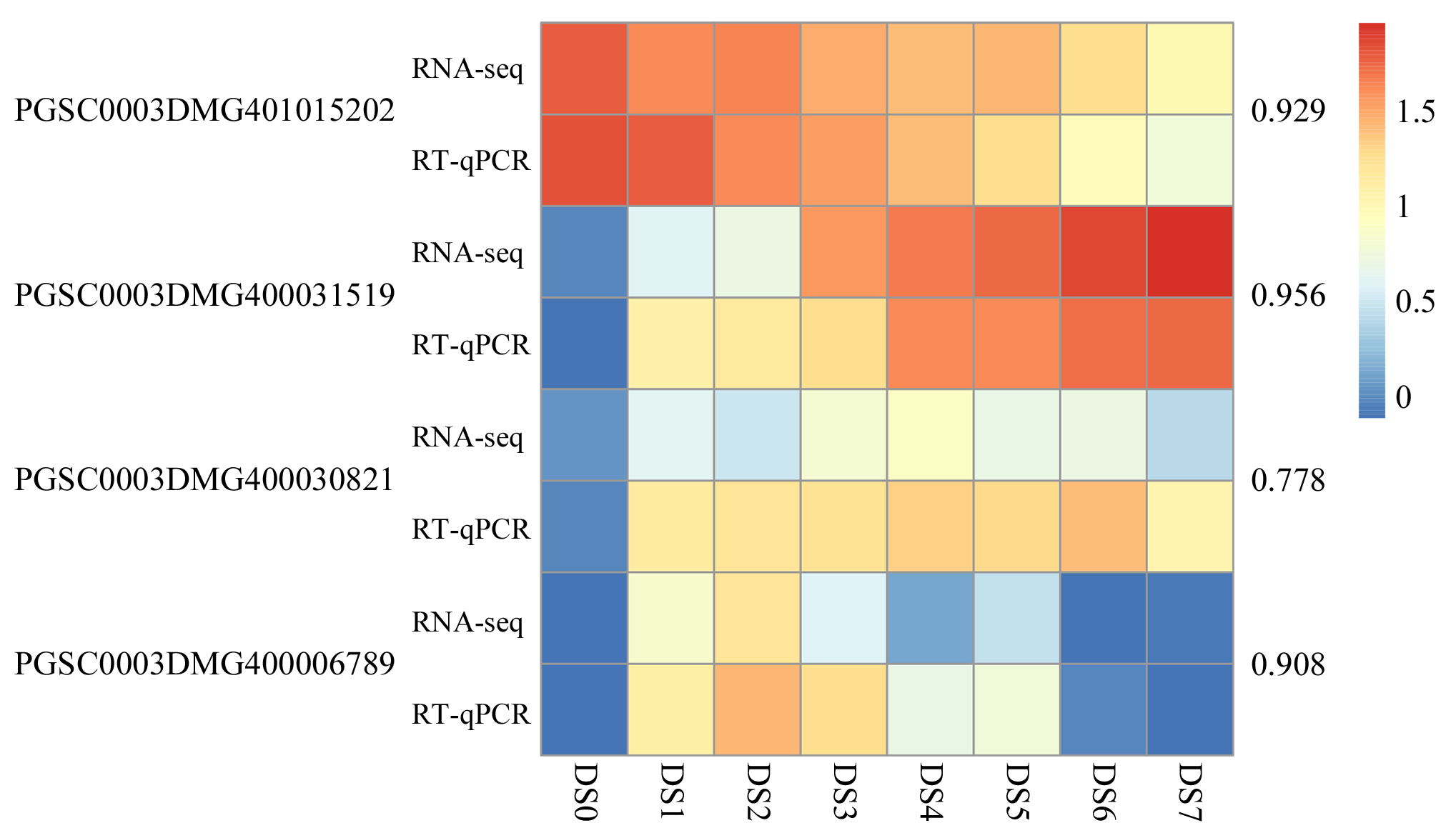

Supplement: Supplementary Figure S5 — RT-qPCR validation of RNA sequencing results. Four hub genes with the highest connectivity in four modules were selected for the confirmation of differentially expressed genes using the same RNA samples that were used for RNA sequencing. Pearson correlation between the gene expression levels measured using RT-qPCR and RNA-Seq was used for validation. The colors in the heat plot represents values for log2 fold change for each sample. [file Image_5.TIF]
